# Aspirin inhibits human telomerase activation in unstable carotid plaques

**DOI:** 10.3892/etm.2013.1082

**Published:** 2013-04-29

**Authors:** FANGMING LI, YI GUO, XIN JIANG, JIANXIN ZHONG, GUANDONG LI, SHENGGANG SUN

**Affiliations:** 1 Department of Neurology, Jiangmen Central Hospital, Jiangmen, Guangdong 529070;; 2 Department of Neurology, The Second Affiliated Hospital of Jinan University, Shenzhen, Guangdong 518020;; 3 Department of Neurology, Union Hospital, Tongji Medical College, Huazhong University of Science and Technology, Wuhan, Hubei 430022, P.R. China

**Keywords:** aspirin, telomerase activation, unstable carotid plaques

## Abstract

The activation of telomerase in unstable plaques is an important factor in atherosclerosis, and may be predictive of the risk of cerebrovascular diseases. Human telomerase reverse transcriptase (hTERT) is a subunit of telomerase that is essential for telomerase activation. The aim of the present study was to investigate whether aspirin inhibits the activation of telomerase and hTERT in unstable carotid plaques. Polymorphonuclear neutrophils (PMNs) derived from carotid plaques were isolated from the washing medium of angioplasty balloons, while circulating PMNs, isolated from arterial blood, served as the controls. A polymerase chain reaction-based telomeric repeat amplification protocol (TRAP) enzyme-linked immunosorbent assay (ELISA) was used to measure the telomerase activity in the cells following treatment with aspirin. The mRNA and protein expression of hTERT were detected by a reverse transcription-polymerase chain reaction (RT-PCR) and western blot analysis, respectively. The results revealed that the atherosclerotic plaques were positive for telomerase activity, and that aspirin inhibited the telomerase activity of the PMNs derived from the plaques. In addition, aspirin was demonstrated to inhibit the mRNA and protein expression of hTERT through the suppression of hTERT transcriptional activity; however, it had no inhibitory effect on the telomerase activity of the circulating PMNs. Thus, the activation of telomerase in resident PMNs is critical in the instability of carotid plaques. The upregulation of telomerase and hTERT during the progression of atherosclerosis may indicate a role for telomerase in the vascular remodeling that occurs during atherogenesis. Aspirin was demonstrated to inhibit the activation of telomerase via an hTERT-dependent manner in the PMN cells of unstable carotid plaques, and thus hTERT may be considered as a target in the treatment of cerebrovascular diseases.

## Introduction

Telomeres are specialized deoxyribonucleic acid (DNA)-protein structures that contain non-coding TTAGGG repeats and telomere-associated proteins, and are essential for chromosome stability. Telomere length is an indicator of replicative history, and has been considered a biomarker of aging (1). The maintenance of telomeres is primarily achieved by telomerase, a ribonucleoprotein with reverse transcriptase activity that uses its internal ribonucleic acid component as a template for the synthesis of telomeric DNA. Telomerase activity is present during early development and in adult germline and stem cells of self-renewing tissues; however, it is absent or functionally insufficient in adult somatic cells. Human telomerase reverse transcriptase (hTERT) is a subunit of telomerase, and is essential for telomerase activity (2,3). In the absence of hTERT, telomeres undergo shortening with cell division, a process that may act as a mitotic clock and trigger entry into senescence. The shortening of telomeres is thus considered to be responsible for the limited lifespan of somatic cells in culture, and has also been correlated with organismal aging (4–6). The mechanisms by which telomerase controls these processes are beginning to be understood, and include effects on the signaling cascades that regulate apoptosis. The inhibition of telomerase and the ensuing shortening of telomeres below a critical length may result in apoptosis in various cell types, whereas the induction of telomerase activity is correlated with a resistance to apoptosis (7,8). In particular, polymorphonuclear neutrophils (PMNs) have a finite lifespan, and typically die by undergoing apoptosis. Thus, PMN apoptosis represents a control mechanism limiting the toxic potential of these short-lived, terminally differentiated cells (9,10). Post-mortem studies have revealed the infiltration of PMNs in unstable atherosclerotic plaques, suggesting that PMNs may be involved in plaque destabilization (11). Classical non-steroidal anti-inflammatory drugs (NSAIDs), including aspirin, exert chemopreventive effects on atherosclerosis. Animal, epidemiological and clinical studies have indicated that aspirin may significantly reduce the risk of atherosclerosis, and predict the risk of cerebrovascular diseases, due to its inhibition of cell proliferation and angiogenesis (12,13). Although the anti-atherosclerotic mechanisms of aspirin have been extensively studied, little is known with regard to the interrelation between aspirin and the telomerase activity in PMNs. Therefore, the aim of this study was to determine whether aspirin inhibited hTERT and telomerase activity in unstable carotid plaques.

## Materials and methods

### Design and subjects

Ten patients with lipid-rich plaques and severe (>70%) internal carotid artery (ICA) stenosis underwent carotid angioplasty and stenting procedures in the Department of Neurology, Jiangmen Central Hospital (Jiangmen, China). Patients with chronic or acute infections, or an inflammatory condition, as defined elsewhere (14), were excluded from the study.

There study included seven males (70%) and three females (30%), with a mean age of 73±10 years (range, 47–75 years). Concomitant risk factors, such as hypertension, coronary artery disease, diabetes and a history of tobacco use, were present in 80, 50, 40 and 30% of patients, respectively. The protocol was approved by the Ethics Committee of Jinan University (Shenzhen, China), and written informed consent was obtained from all patients.

### Isolation of PMNs from ICA plaques, cell culture and aspirin treatment

PMNs were isolated from carotid atherosclerotic plaques with a novel approach, as described in a previous study by Narducci *et al* (15). Following carotid angiography, all patients underwent carotid artery balloon angioplasty and stenting (CBAS) in the culprit stenosis. Heparin (3,000 IU) was administered to all patients. The stent deployment was preceded by predilation with Maverick angioplasty balloons (Boston Scientific, Natick, MA, USA). In brief, the PMNs were collected as follows: the predilation balloon was inflated for 20 sec at a mean of 10 atmospheres (range, 6–14 atmospheres). The balloon was then deflated and the guiding catheter was immediately retracted. The washing medium was collected into tubes, and was then supplemented with 5 ml Cytolyt^®^ solution (Cytyc Corporation, Boxborough, MA, USA). The balloon remained inside the guiding catheter for <5 sec, in order to minimize the contamination by blood. The washing medium was centrifuged at 1,200 × g for 10 min, and the cell pellet was then collected and added to 20 ml Preservcyt^®^ solution (Cytyc Corporation). The cells were maintained at 37°C in an atmosphere with 5% CO_2_ and 95% air, in RPMI-1640 medium containing 10% fetal bovine serum (FBS; Gibco^®^, Invitrogen Life Technologies, Carlsbad, CA, USA), 100 U/ml penicillin and 100 *μ*g/ml streptomycin. Following treatment with aspirin (0.5 mM) (16) for 48 h, the cell lysate was collected for the detection of telomerase activity. Cells treated with dimethyl sulfoxide (DMSO) served as a control, and the final concentration of DMSO was ≤0.1%. Data are expressed as the mean ± standard deviation from three independent experiments.

### Peripheral cell isolation

During the carotid angiography, blood was sampled from the right femoral artery. The PMNs were isolated with Polymorphprep™ separation medium (Nycomed Pharma AS, Oslo, Norway) and, following centrifugation at 500 × g for 30 min at 20°C, the PMNs were collected and washed twice in phosphate-buffered saline (PBS). Contaminating erythrocytes were removed by hypotonic lysis. The PMNs were stored at −80°C.

### Detection of telomerase activity

The activity of telomerase was analyzed with a TRAPeze^®^ Telomerase Detection kit (Intergen Co., Oxford, UK), in accordance with the manufacturer's instructions. In brief, 50 ng cell extract was used in a polymerase chain reaction (PCR) assay, prior to 5 *μ*l PCR product being used in an enzyme-linked immunosorbent assay (ELISA). The absorbance was measured at 450 and 690 nm using a microplate reader (Bio-Rad Laboratories, Inc., Hercules, CA, USA).

### Reverse transcription (RT)-PCR

Total RNA was extracted from the PMN cells using TRIzol^®^ Reagent (Invitrogen Life Technologies), and the cDNA was synthesized using a Thermoscript RT-PCR System kit (Invitrogen Life Technologies). The PCR primers were as follows: hTERT, forward: 5′-CGG AAG AGT GTC TGG AGC AA-3′ and reverse: 5′-GGA TGA AGC GGA GTC TGG A-3′; and GAPDH, forward: 5′-GAC CAC ACG CCA TGC CAT CAC-3′ and reverse: 5′-GTC CAC CAC CC TG TTG CTG TA-3′. A total of 30 cycles of PCR were completed for hTERT and 28 cycles for GAPDH (94°C for 1 min, 55°C for 1 min and 72°C for 1 min), and the expression of hTERT was normalized to that of GAPDH.

### Western blot analysis

The extracted proteins (60 *μ*g) were subjected to sodium dodecyl sulfate-polyacrylamide gel electrophoresis (SDS-PAGE), and were then transferred to a nitrocellular membrane. hTERT and actin were detected with hTERT and actin polyclonal antibodies, respectively (Santa Cruz Biotechnology, Inc., Santa Cruz, CA, USA). Actin served as an internal control.

### Statistical analysis

Statistical analysis was performed using Excel software (Microsoft Corporation, Redmond, WA, USA). The Student's t-test was used to compare data between two groups. Data are expressed as the mean ± standard deviation (SD). P<0.05 was considered to indicate a statistically significant difference.

## Results

### Effect of aspirin on telomerase activity

**Figure 1.**
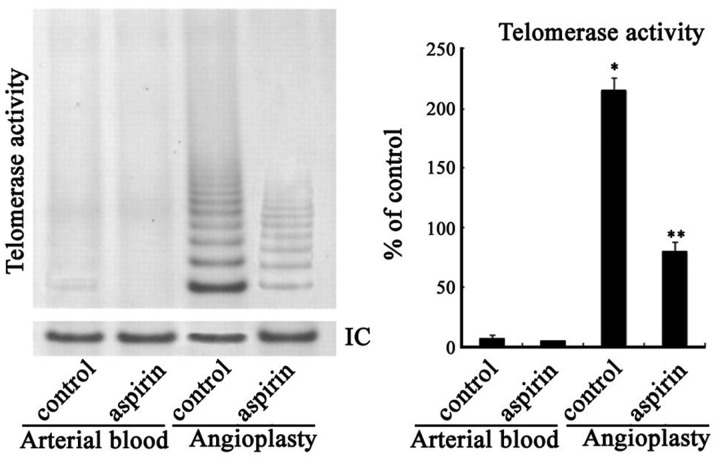
Telomerase activity in unstable carotid plaques. Extracts of polymorphonuclear neutrophils (PMNs) isolated from arterial blood and from the washing medium of angioplasty balloons, derived from unstable carotid plaques, underwent telomerase activity detection by a telomeric repeat amplification protocol assay, in the presence of an internal control (IC). Telomerase activity was undetectable in the PMNs from the arterial blood; however, in the same patients, the telomerase activity was significantly increased in the PMNs from the washing medium. Aspirin inhibited the telomerase activity in the PMNs of unstable carotid plaques, but not in the circulating PMNs. Cells treated with dimethyl sulfoxide (DMSO) served as controls. ^*^P<0.001 vs. PMNs from arterial blood; ^**^P<0.001 vs. control.

The activity of telomerase, measured by a telomeric repeat amplification protocol (TRAP) assay, in the PMNs isolated from the arterial blood and from the washing medium of the angioplasty balloons, is displayed in Fig. 1. Telomerase activity was observed to be significantly increased in the PMNs isolated from the angioplasty washing medium compared with the arterial blood control. Telomerase activity was undetectable in the PMNs derived from the arterial blood of patients with CBAS.

Telomerase activity was higher in the PMNs from the unstable carotid plaques than in the circulating PMNs. Aspirin (0.5 mM) inhibited the telomerase activity of the plaque-derived PMNs by ∼62.8% compared with that of the PMNs treated with DMSO (79.5 versus 213.8% activity, for aspirin and DMSO-treated cells, respectively). However, aspirin did not exert any inhibitory effect on the telomerase activity in the circulating PMNs.

### Effect of aspirin on the mRNA and protein expression of hTERT in unstable carotid plaques

To investigate whether aspirin was able to inhibit the mRNA and protein expression of hTERT, RT-PCR and western blot analysis were performed. Extracts of PMNs isolated from the angioplasty washing medium demonstrated high levels of mRNA and protein expression of hTERT in the unstable carotid plaques. However, the levels of mRNA and protein expression of hTERT were almost undetectable in the PMNs from the arterial blood of patients who underwent CBAS.

**Figure 2.**
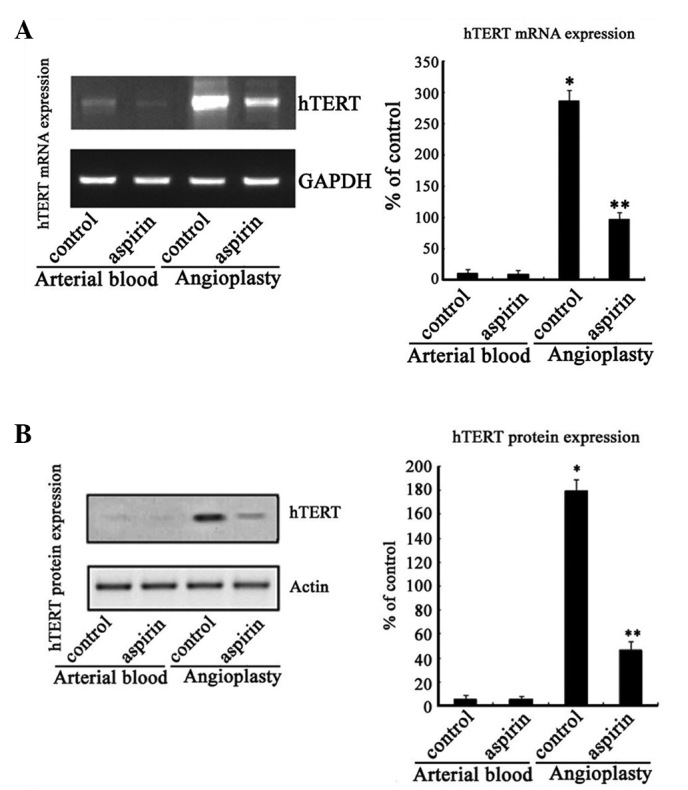
Effect of aspirin on the mRNA and protein expression of human telomerase reverse transcriptase (hTERT) in unstable carotid plaques. (A) Reduced hTERT mRNA expression in aspirin-treated polymorphonuclear neutrophils (PMNs), as demonstrated by reverse transcription-polymerase reaction (RT-PCR). (B) Reduced hTERT protein expression in aspirin-treated PMNs, as demonstrated by Western blot analysis. The mRNA and protein expression of hTERT remained unchanged in circulating PMNs. Cells treated with dimethyl sulfoxide (DMSO) served as controls. ^*^P<0.001 vs. PMNs from arterial blood; ^**^P<0.001 vs. control.

The mRNA expression of hTERT was inhibited by 66.2%, following aspirin (0.5 mM) treatment, compared with the cells treated with DMSO (96.7 versus 286.1% expression for aspirin-treated and control cells, respectively; Fig. 2A). Aspirin treatment (0.5 mM) inhibited the protein expression of hTERT by 73.8% compared with the cells treated with DMSO (46.7 versus 178.3% expression for aspirin-treated and control cells, respectively; Fig. 2B). However, aspirin did not exert any inhibitory effects on the mRNA and protein expression of hTERT in the circulating PMNs (Fig. 2).

## Discussion

The activation of telomerase, which occurs in the majority of atherosclerotic plaques, results in the prolonged lifespan of cells. This is an important feature in the early phases of instability of atherosclerotic plaques (17–19). However, there have only been a limited number of studies that have investigated the effect of aspirin on the telomerase activity in PMNs. In order to characterize the mechanism of delayed PMN apoptosis in unstable carotid plaques, and the correlation between aspirin and telomerase activity, the activity of telomerase was determined in PMNs isolated from peripheral blood in patients with lipid-rich plaques and severe (>70%) ICA stenosis. For the same patients, telomerase activity was also measured in PMNs isolated from the washing medium used during percutaneous angioplasty and stenting procedures. In addition, the telomerase activity, and mRNA and protein expression of hTERT were determined in cells that had receieved aspirin treatment. TERT possesses the catalytic activity of telomerase, and is primarily regulated through transcriptional mechanisms (20).

The present study demonstrated that there was high telomerase activity in the PMNs from the carotid plaques of patients with severe ICA stenosis, but not in the PMNs from peripheral blood. Notably, in patients with severe ICA stenosis, the sole predictor of telomerase activity in the coronary atherosclerotic plaques was a reduced time interval from the onset of the symptoms to PMN collection, which supports the potential importance of telomerase reactivation in PMN persistence during the early phases of carotid plaque instability.

Under normal circumstances, PMNs, similar to other somatic cells, divide a limited number of times, prior to entering a nondividing state, known as replicative senescence. In general, telomerase is inhibited in inflammatory cells (21,22). The present study revealed that telomerase activity was virtually undetectable in the circulating PMNs, and that the reactivation of telomerase in these cells was possible. This reactivation represents a a method of overcoming replicative senescence (8,23). The high telomerase activity in the PMNs from the carotid atherosclerotic plaques in the current study suggested that there was a local process leading to the reactivation of the intracellular enzyme, which resulted in the prolonged survival of these inflammatory cells, as observed in cells retaining a high proliferative potential (24,25). Furthermore, the telomere dynamics and the changes in telomerase activity were consistent with a proliferative state. A highly specific correlation and an early causal interrelation have been observed between telomerase activation and indefinite cell proliferation (26,27).

Therefore, it was indicated that in the patients with severe ICA stenosis and unstable plaques, the survival of local activated PMNs was prolonged due to the reactivation of telomerase. It was likely that this exacerbated the tissue damage and oxidative stress due to PMN activation, and maintained the active inflammatory process, since neutrophil apoptosis has been identified as a crtitical mechanism in the cessation of inflammation. However, as telomerase reactivation appears to be important in delaying apoptosis and inducing the growth of cancer cells (28), this intracellular mechanism may have also prolonged the activation of the PMNs in the carotid plaques by extending their local lifespans.

Classical NSAIDs, including aspirin, have been demonstrated to exert chemopreventive effects on atherosclerosis. The present study revealed that aspirin inhibited the telomerase activity in PMNs from carotid lipid-rich plaques, but had no inhibitory effect on the circulating PMNs. Further studies were performed to determine whether the inhibition of telomerase by aspirin was attributed to the inhibition of hTERT mRNA and protein expression. RT-PCR and western blot analysis demonstrated that aspirin reduced the mRNA and protein expression levels of hTERT in the PMNs from carotid plaques, but not in circulating PMNs. This may be attributed to the altered catalytic activity of the telomerase holoenzyme following aspirin treatment.

Previous studies have observed that the phosphorylation of hTERT is involved in the regulation of telomerase (16), and that certain kinases, including Akt kinase and protein kinase C (PKC), mediate the phosphorylation of hTERT, leading to telomerase activation (29,30). Aspirin may downregulate the expression of telomerase by interfering with these pathways. It is likely that aspirin inhibits the transcriptional activity of hTERT by suppressing the activation or binding activity of the previously mentioned factors. In the present study, the inhibitory effect of aspirin on hTERT was correlated with the inhibition of telomerase activity, which suggests that the decreased hTERT expression accounts for the inhibition of telomerase activity following aspirin treatment.

In conclusion, aspirin is able to inhibit telomerase activity, primarily through the suppression of transcriptional activity, and the mRNA and protein expression of hTERT in the carotid atherosclerotic plaques. The promoter (−145 to −330 bp) of hTERT may be the cis-response element to aspirin. Telomerase activity is an important factor in unstable plaques, and is predictive of the future occurrence of cerebrovascular diseases. Thus, an enhanced understanding of the mechanisms involved in these processes is required to enable the application of telomerase or telomerase-related compounds as targets in the treatment of cerebrovascular diseases. Further studies are required to identify the potential cis-response elements to aspirin.
